# Updating ‘Stories’ on social media and its relationships to contextual age and narcissism: A tale of three platforms – WhatsApp, Instagram and Facebook

**DOI:** 10.1016/j.heliyon.2022.e09412

**Published:** 2022-05-14

**Authors:** Devadas Menon

**Affiliations:** IGNOU University, Ahmedabad 380058, Gujarat, India

**Keywords:** Social media stories, Status update, Uses and gratifications, WhatsApp, Instagram, Facebook, Contextual age, Life position indicators, Narcissism

## Abstract

This study extends the uses and gratifications research into the feature use of social media platforms by identifying the motives for updating ‘Stories’ on three social media platforms, i.e. WhatsApp, Instagram and Facebook. Using survey data from adults (N = 338), this research identified seven motivations (i.e. *socially rewarding self-promotion, social sharing, social influence, disclosure, escape, entertainment, and trendy fashion*) for updating Stories on WhatsApp, Instagram and Facebook. Age and gender differences were identified in the motivations for updating Stories on the three platforms. Cross-platform analysis revealed significant differences in the motivations for updating Stories across the three platforms. Social influence and disclosure motives positively predicted the intensity of updating Stories on all three platforms. Socially rewarding self-promotion predicted the intensity of updating Stories on Instagram and Facebook. Social sharing positively predicted the intensity of updating Stories on WhatsApp and Instagram, and trendy fashion emerged as a positive predictor of story updates only on Instagram. Finally, an analysis of social and psychological predictors revealed that Interpersonal interaction and Social activity positively predicted the intensity of updating Stories across the three platforms. Narcissists also exhibited high intensity of updating Stories on WhatsApp, Instagram, and Facebook.

## 1 Introduction

Are you at the pinnacle of happiness? or sailing through the difficult moments in your life? ‘Story’, a ubiquitous feature hiding inside the social media platforms, let you share the reflections of your mind instantly with your social circle. “Share your everyday moments! (Instagram) Social media platforms call people to express their feelings and emotions through the ‘Stories’ feature, which has a 24 h life span. From the quotes of John Lennon to the scenes of the web series binge-watched last night, people share anything as ‘Stories’. The feature originally introduced by Snapchat today has had 1.5 billion daily users across all the platforms (Ho, 2019). Besides its use as a personal communication feature, Stories today are used as an effective marketing tool by commercial and business firms.

‘Stories’ are a feature on social media platforms that allow users to create a time-sensitive and auto-playing cacophony of images, videos, or both that can be enriched with backgrounds, texts, stickers, animations, music, emojis and effects. Social media Stories are designed to be alive for just 24 h, and after that, they disappear automatically. This Story feature's main aim is to instantly tell others what is happening in one's life or business in the form of a story. Stories appear as gradient borders around the users' profile pictures on their social media platforms. Story features on some platforms let users tagging, add locations and apply hashtags. Stories feature designed exclusively for vertical viewing to fit in smartphones. According to the Mobile Overview Report (MOVR 2014), smartphone users hold their phones vertically about 94% of the time. Further recent studies (Omar and Dequan, 2020; Scherr and Wang, 2021; Yoon et al., 2021) have identified that short mobile videos captivate users' attention and successfully engage them.

The first social media platform to introduce the Story feature in 2013 was Snapchat (O'Connell, 2020). This feature of Snapchat enables the users to arrange snaps in chronological order to tell a story, and these stories will be automatically deleted after 24 h. Seeing this feature's popularity among youngsters, particularly millennials and Gen Xers, other social media platforms started mimicking this feature. In 2015 twitter introduced this feature and named it ‘moments’. Instagram launched this feature in 2017 and called this feature ‘Story’. In 2017 both Facebook and WhatsApp started providing the feature of ‘Story’ to their users. WhatsApp introduced this feature as ‘status Stories’ and later renamed it ‘Status’ (Olson, 2017). Today users of various social media platforms call this feature with 24 h life span designed to create a vertical audiovisual collage as either ‘Story’ or ‘Status’.

The transient nature of Stories was what made them popular among youngsters. According to Gotter (2020), the temporary nature of Stories (the fear of missing out, FOMO) creates a sense of immersion and scarcity that keeps the users in the loop. People and businesses update Stories on social media platforms to provide their followers with a glimpse of what's happening in their lives at the moment. The recent statistics showed that Stories have momentum across all the social media platforms. According to recent reports, Stories on social media platforms grew 15X faster than feeds from Q2 2016 to Q3 2017 (Constine, 2018). Among the platforms, WhatsApp has 450 Million daily Story users, followed by Instagram with 300 Million and Facebook with 70 Million (Constine, 2018). Although the popularity of Stories is expanding day by day, the related research has not yet grown proportionately.

Looking into the immense popularity of the Stories feature, the present study aims to determine the various motivations behind updating Stories on social media platforms. Besides identifying the motivations, the current study attempts a cross-platform analysis of Stories on the three most popular social media platforms, i.e. WhatsApp, Instagram, and Facebook, to understand better the uniqueness of updating Stories on each platform. In addition to this, this research also examines the influence of demographic variables on updating Stories. Guided by the past studies on social media platforms (Sheldon and Bryant, 2016; Davenport et al., 2014; McCain and Campbell, 2018; Meng and Leung, 2021), we also tried to locate the relationship between social and psychological predictors (i.e. contextual age and narcissism) and Stories. To address the aims of the study, the theoretical framework of Uses and gratifications has been utilised. Before moving on to the study's theoretical framework, the next section explains the scope and rationale of the study.

### 1.1 Scope and rationale

As mentioned in the previous section, although Stories are gaining immense popularity day by day, no research has been conducted yet to identify the exact motives behind their usage and popularity. Besides its use as an interpersonal communication medium to express feelings and emotions, Stories are widely used today as a social media marketing tool by corporate firms, PR agencies, celebrities and social media influencers. Hence a systematic study behind the phenomenon of social media Stories is highly warranted. Such a study can provide a kaleidoscopic view of the know-how and nuances of this new yet highly popular social media feature. Second, besides its practical implications, we also think this study will contribute to the growing body of Uses and gratifications research by extending the theory to Stories, a particular feature on social media platforms. Lastly, literature is scarce on cross-platform analysis of social media platforms. To the best of our knowledge, only very few studies, for example; Alhabash and Ma (2017); Sheldon et al., (2021a), (2021b), were conducted to analyse the cross-platform difference among social media platforms. The current study addresses the existing research gap by examining the cross-platform differences in updating Stories on WhatsApp, Instagram and Facebook.

We have selected our sample from India because of two main reasons. First, India is one of the top countries in terms of social media usage, with 518 million users in 2020. The market is expected to grow by 1.5 billion by 2040 (Keelery, 2021b, August 17). India ranks first globally in terms of Whatsapp, Facebook and Instagram usage, with 346 million, 340 million, and 180 million users, respectively (Statista, 2021, September 10). Hence in the light of these statistics, India seems to be the ideal country to study the Stories update on the three platforms. Second, by choosing samples from India, this study seeks to address the argument of Ruggiero (2000) that the ‘Uses and Gratification theory has limited acceptability and popularity outside the United States’.

## 2 Theoretical framework and research questions

### 2.1 Uses and gratifications theory

The media usage behaviour of individuals is guided by the needs that they seek to gratify from the particular media (Katz et al., 1974; He et al., 2021). Uses and gratification theory is one of the media use theories widely utilised by researchers to identify the motives behind using a particular media (Whiting and Williams, 2013; Parmelee and Roman, 2019; Bucknell Bossen and Kottasz, 2020; Scherr and Wang, 2021). The uses and gratification theory characterises users as active, discerning, and guided by certain specific motivations in their media selection and usage (Levy, 1984, Perse and Rubin, 1988; Timmermans and De Caluwé, 2017; Steiner and Xu, 2020). ‘Motivations are general dispositions that influence people's actions taken to fulfil a need or a want (Papacharissi and Rubin, 2000, p.179, p.179)’. The uses and gratification theoretical framework is designed to capture the motivations behind using a particular media and understand user behaviour, outcomes and perceptions (Katz et al., 1974; Meng and Leung, 2021). Besides motives of media usage, social and psychological factors also influence particular media's selection, usage, and behaviour (Papacharissi and Rubin, 2000; Sheldon and Bryant, 2016; Meng and Leung, 2021). Uses and gratifications theory provides a cutting edge, inclusive and parsimonious framework to tap people's media usage behaviour.

In the last fifty years, the uses and gratifications theory has undergone many conceptual refinements and evolved into a cutting edge framework by understanding audience gratifications behind the usage of traditional media such as newspapers, radio, television, and new media such as the internet and various social media (Sundar and Limperos, 2013; Ray et al., 2019; Meng and Leung, 2021). The advancement in ICT and the emergence of social media platforms have restructured and reconfigured the nature of gratifications (Sheldon et al., 2017), warranting further advancement in uses and gratifications theory (Alhabash and Ma, 2017). Scholars have addressed these challenges by incorporating traditional and new gratifications emerging from new media (Sundar and Limperos, 2013). Nevertheless, the uses and gratifications theory has the potential to evolve into a sophisticated model, which can help the researchers add more nuanced motives, social and psychological antecedents, and media usage behaviour to the theory (Ruggiero, 2000). In the light of evolving new media, Sundar and Limperos (2013) classified uses and gratifications broadly under two categories: user-centred conceptualisations (like entertainment, escape etc.) and platform-based affordances (such as modality, agency, interactivity and navigability). In the last ten years, a handful of studies have successfully captured the gratifications emerging from the social media platform with a modernised U&G framework (Meng and Leung, 2021).

We have utilised the uses and gratifications theory as our guiding framework for at least three reasons. First uses and gratifications theory provided a cutting edge theoretical framework to locate the gratification motives in the initial stages of each new communication media (Ruggiero, 2000; Sundar and Limperos, 2013). Secondly, the Uses and gratification theory allows the researcher to use a holistic approach by incorporating qualitative and quantitive methods; for example, Ray et al. (2019) used a two-step procedure to identify the motives for using food delivery apps. Thirdly the dependence on the uses and gratifications approach to identify the motivations behind updating Stories recognises the interactive nature of the Story feature (Katz et al., 1974, Ruggiero, 2000; Sundar and Limperos, 2013; Rathnayake and Winter, 2018).

The various uses and gratifications studies pertaining to the motivations behind using various social media platforms are summarised below.

### 2.2 Uses and gratifications and social media platforms

Stories on social media are a particular feature or platform-centred modality affordance (Sundar and Limperos, 2013; Rathnayake and Winter, 2018) than a new application. No previous study has identified the uses and gratifications of this new feature. Moreover, when social media platforms include additional features, there is an increased need to analyse the uses and gratification of the particular feature (Smock et al., 2011; Wohn et al., 2011; Karnik et al., 2013). Applying the Uses and Gratification theory to a particular feature can elicit gratification obtained from the feature, user engagement and their level of participation (Smock et al., 2011; Baek et al., 2011). Hence, it begs whether we should use an existing set of gratification motives from other social media platforms to identify the motivations behind updating Stories or to extent.

Numerous prior studies have focused on particular features of social media, such as engaging in Facebook groups (Park et al., 2009), sharing links on Facebook (Baek et al., 2011), playing SNS games (Lee et al., 2012; Sheldon, 2014), participating in music video sharing Facebook groups (Karnik et al., 2013), Facebook music listening applications (Krause et al., 2014), sharing links (Baek et al., 2011), photo sharing (Oeldorf-Hirsch and Sundar, 2016; Malik et al., 2016), photo tagging (Dhir et al., 2017), hashtagging (Erz et al., 2018; Sheldon et al., 2020), and usage of WhatsApp Stickers (Al-Maroof et al., 2021). Although certain new feature centred gratifications have emerged from the studies, the results of these studies revealed a considerable amount of overlap in usage motivations. The identified common gratifications across these features included entertainment, social interaction, escape and pass time were mirror motivations for more general usage of social media platforms (for example, Joinson, 2008; Quan-Haase and Young, 2010; Whiting and Williams, 2013). Further, Sundar & Limperos (2013) analysed the gratifications obtained from various media from the 1940s to 2013 and observed commonalities across various media in certain gratifications such as entertainment, escape, relaxation and information seeking. Sundar and Limperos (2013) suggested that integrating traditional gratifications and platform centred affordances is the most parsimonious way to tap the potential gratifications from new media. Based on these assumptions from prior literature, we have analysed the past uses and gratifications studies related to the three platforms under study (i.e. WhatsApp, Instagram and Facebook) and Snapchat, as the feature of Story is borrowed from Snapchat.

Although WhatsApp is one of the most popular instant messaging applications, scanty literature on its usage motivations is available. The available literature on the uses and gratifications of WhatsApp revealed the following motives for its usage: information seeking (Islam et al., 2020), entertainment (Kircaburun et al., 2020; Islam et al., 2020; Arruda Filho and Ferreira, 2021), social interaction (Awaf, 2015; Karapanos et al., 2016; Bautista and Lin, 2017; Arruda Filho and Ferreira, 2021), community building (Kircaburun et al., 2020; Arruda Filho and Ferreira, 2021), socialisation (Awaf, 2015; Bautista and Lin, 2017; Islam et al., 2020; Kircaburun et al., 2020), habit (Islam et al., 2020), trendiness (Awaf, 2015), social influence (Karapanos et al., 2016), and catharsis (Bautista and Lin, 2017).

The main motives identified by recent uses and gratification research on Facebook can be summarised as information sharing, social interaction, social influence, habitual past time, entertainment, relaxation, disclosure, cool and new trend and receiving recognition from others (Papacharissi and Mendelson, 2010; Hollenbaugh and Ferris, 2014; Oeldorf-Hirsch and Sundar, 2016; Malik et al., 2016; Lin and Chu, 2021; Menon and Meghana, 2021). Similarly, Instagram studies (Sheldon and Bryant, 2016; Alhabash and Ma, 2017) revealed that gratifications sought by people were surveillance, documentation, coolness, creativity, entertainment, convenience, medium appeal, passing time, self-expression, social interaction, and information sharing. As for Snapchat, the research found that entertainment, medium appeal, convenience, passing time, self-expression, self-documentation, information seeking and social interaction (Alhabash and Ma, 2017).

Alhabash and Ma (2017) conducted a cross-platform analysis of four social media platforms, i.e. Facebook, Instagram, Snapchat and Twitter and found that all the motivations except information sharing were significantly different across the four platforms. For example, the primary motive for using Facebook was convenience, whereas entertainment was the primary motive for the other three platforms.

As summarized above, several studies are available on WhatsApp, Instagram, and Facebook usage motivations and their features like photo sharing, photo tagging and hashtagging. But although updating Stories on social media platforms is exceedingly popular among people, no study has yet identified the motivations behind this activity. To bridge this research gap, we pose the following research questions in the context of the current research.

***RQ1***. What are the motivations for updating Stories on WhatsApp, Instagram and Facebook?

***RQ2***. What are the differences (if any) in motivations for updating Stories on WhatsApp, Instagram, and Facebook?

### 2.3 Gender and gratification motives

There is a substantial body of literature examining age and gender differences in usage behaviour with reference to the internet. Gender differences concerning social media platforms have also been actively investigated (Hargittai, 2007; Muscanell and Guadagno, 2012; McAndrew and Jeong, 2012; Tifferet and Vilnai-Yavetz, 2014; Dhir and Torsheim, 2016; Scherr and Wang, 2021). Some of these studies (Joiner et al., 2012; Muscanell and Guadagno, 2012) identified gender as a significant predictor of using a specific feature or activity. For instance, Malik et al. (2016), in their study on digital photo sharing on social media, found that male users seek more habit and disclosure motivation than female users. Similarly, studies (Hollenbaugh and Ferris, 2014; Dhir and Torsheim, 2016) have found that age differences exist in the motivation to use various social media. In recent research on TikTok usage behaviour, Scherr and Wang (2021) found that trendiness increases and escapism decrease with age. Since Stories are a new feature embedded on social media platforms, we believe there will be substantial age and gender differences in its usage motivations. Thus we pose the following research question.

***RQ3***. Do the motives for updating Stories on Whatsapp, Instagram, and Facebook depend on the age and gender of its users?

### 2.4 Social and psychological predictors

The previous research supports the conceptualisation of a hybrid approach to eliciting gratifications obtained from new media technologies. Hence researchers (Papacharissi and Rubin, 2000; Sheldon and Bryant, 2016) have used social and psychological antecedents and user motivations to predict a particular media behaviour. Contextual age and narcissism were two important social and psychological antecedents used by researchers to predict the user engagement behaviour and intensity of social media platforms (Sheldon and Bryant, 2016).

*Contextual age:* Contextual age, also known as life position indicators, was introduced by Rubin and Rubin (1981) to overcome the limitations of chronological age in mass media research. Rubin and Rubin's (1981) conceptualisation of life position indicators originally contained six components: interpersonal interaction, social activity, life satisfaction, mobility, health and economic security, which are found to be influencing media usage behaviour (Rubin and Rubin, 1982). Papacharissi and Rubin (2000), in their study on the internet, observed that those who were less satisfied with their life used the internet as a functional alternative for interpersonal interaction to pass the time. Contextual age was also used in social media research. Sheldon (2014) examined the relationship between contextual age and motives for playing Facebook games and found that people who were less satisfied spent more time on Facebook games and played more often. In another study, Sheldon and Bryant (2016) found that life satisfaction has negatively predicted the intensity of Instagram usage. Analysis of this past literature indicates that contextual age can also influence updating Stories.

*Narcissism:* Narcissists are termed as people having a self-fixation, a high level of self-importance, and a strong desire to be admired (Buffardi and Campbell, 2008; Sheldon and Bryant, 2016). Narcissists think that they are unique, special and superior to others (Sheldon and Bryant, 2016). Narcissists prefer to have a shallow relationships on social media platforms and always try to have full control over their self-presentation (Manago et al., 2008; Sheldon and Bryant, 2016). Several studies have found that narcissists exhibit a higher degree of disclosure and more interest in sharing photos and videos on online platforms. Narcissists showed more interest in posting personal photos on Facebook and showed greater interest in liking and commenting on the photographs uploaded by others (Sheldon and Bryant, 2016). When it comes to Instagram, Sheldon & Bryant (2016), found that narcissism was positively related to the usage of Instagram to be cool and surveillance of others. Considering the lack of literature on the influence of contextual age and narcissism on updating Stories on WhatsApp, Instagram and Facebook, we ask the following research question:

***RQ3***. How do contextual age and narcissism predict the intensity of updating Stories on WhatsApp, Instagram and Facebook?

## 3 Methodology and research design

### 3.1 Participants and procedure

The data for the current study was gathered from an online survey with a sample (N = 425) of Facebook, WhatsApp, and Instagram users from India who updated their social media ‘stories’ regularly on Facebook, Instagram and WhatsApp. The study approval was obtained from the Ignou University (ID: MHD2002090922), and all participants signed the electronic consent form informing them about the research aim and duration to fill out the questionnaire. Participants were informed about the study objectives and expected benefits, and participation was voluntary. Before filling out the questionnaire, They were also informed that the study might elicit information about their personality, life satisfaction, social activity, and interpersonal interaction. Participants did not receive any compensation for participating in the study. The survey participants were recruited through snowball sampling and completed the survey anonymously via the online web-based platform SurveyMonkey.com. SurveyMonkey platform was commonly used in research, and its validity is supported on par with other online survey tools (Evans et al., 2009; Usry et al., 2018; Brooks et al., 2016). Before conducting the final survey, a focus group interview was conducted with 17 participants who regularly updated their social media Stories on the three platforms. The purpose of this focus group interview was to gather additional information for the design of the final questionnaire, especially to tap the additional gratifications behind updating Stories. The survey was conducted between February to July 2021.

Potential respondents were initially screened through two questions about Story updates: “Do you update Stories on WhatsApp, Instagram and Facebook?”; “Have you updated Stories on WhatsApp, Instagram and Facebook in the last 30 days?”: both responses measured on YES/NO. Those participants who answered YES were qualified to proceed further. A total of 88 participants were excluded from the final sample after various quality checks and convenience for cross-platform analysis, resulting in 338 respondents. All the 338 participants are regular users of Facebook, WhatsApp, and Instagram and regularly update their Stories. Of the 338 participants, 62.1 % were identified as females, and 37.9% were males. The minimum age of the respondent who participated in the survey was 18, and the maximum age was 42 (M_Age_ = 25.5, S. D = 9.08, *Mdn* = 22).

### 3.2 Measures

#### 3.2.1 Contextual age

Rubin and Rubin's (1982) contextual age scale was used to measure the life position indicators comprising the following dimensions: physical health, interpersonal interaction, mobility, life satisfaction, social activity, and economic security. However, considering the statistics of the usage of the three platforms understudy in India (Statista, 2021), physical health, mobility, and economic security were excluded from the study due to the lesser expectation of significant variation within the population. Life satisfaction was measured using three items: “I have been very successful in achieving my aims or goals in life,” “I am very content and satisfied with my life”, “I find a great deal of happiness in my life,” and All the items were measured using a 5-point Likert scale (1 = *Strongly disagree*; 5 = *Strongly agree*). These responses then were averaged and summed into a subscale, and the mean scores of the life satisfaction dimension for Facebook users was 3.51 (S.D = 0.97, Cronbach's α = 0.80), Instagram was 3.51 (S.D = 0.98, Cronbach's α = 0.79) and WhatsApp was 3.53 (S.D = 0.97, Cronbach's α = 0.80). To assess the social activity of the participants, they were asked to express their agreements with the following stated questions on a 5-point Likert scale (1 = *Strongly disagree*; 5 = *Strongly agree*): “I often participate in games, sports or activities with others,” “I often visit with friends, relatives neighbours in their homes”, and, “I often travel, vacation or take trips with others,”. The summation of the average of the responses resulted in the social activity dimension. The social activity dimension of the three platforms under study are as follows, Facebook; Mean = 3.05 (S. D = 1.10 Cronbach's α = 0.75), Instagram; Mean = 3.05 (S. D = 1.10 Cronbach's α = 0.73), and WhatsApp; Mean = 3.11 (S. D = 1.09 Cronbach's α = 0.71). The interpersonal interaction dimension consists of the following three items: “I get to see my friends as often as I would like,” “I have ample opportunity for communication with others”, and “I spend enough time communicating with my family or friends by telephone or e-mail,”. Responses on the 5-point Likert scale were averaged and summed into a subscale. The mean scores of the interpersonal interaction dimension of the three platforms are, Facebook; Mean = 3.47 (S. D = 0.92, Cronbach's α = 0.71), Instagram; Mean = 3.49 (S. D = 0.925, Cronbach's α = 0.73), and WhatsApp; Mean = 3.52 (S. D = 0.89 Cronbach's α = 0.70).

#### 3.2.2 Narcissism

The hypersensitive narcissism scale (Hendin and Cheek, 1997) was used to assess the respondent's covert narcissism score. This scale is considered the most parsimonious and robust measure of narcissism, derived from correlating the items of Murry's (1938) Narcissism scale with an MMPI-based composite measure. Researchers on platforms like Instagram widely used this scale in the recent past (Sheldon and Bryant, 2016). The items on the scale were summed into a subscale for all three platforms separately. The mean score of narcissism for the Facebook users is 2.91 (S. D = 0.72, Cronbach's α = 0.77), Instagram is 2.93 (S. D = 0.73, Cronbach's α = 0.79), and WhatsApp is 2.93 (S. D = 0.70, Cronbach's α = 0.76).

#### 3.2.3 Story update uses and gratifications

A pool of story update gratifications was developed by assembling the measures from past studies on Facebook (Papacharissi and Mendelson, 2010; Quan-Haase and Young, 2010), WhatsApp (Karapanos et al., 2016), and Instagram (Sheldon and Bryant, 2016; Alhabash and Ma, 2017) along with the items that emerged from our focus group interviews. Overall, 36 different reasons for updating Stories were included in the questionnaire. Survey participants had to enter their responses on a 5-point Likert scale (1 = *Strongly disagree*; 5 = *Strongly agree*). Participants had to enter their motivations for updating stories for each platform separately. Three separate sets of exploratory factor analyses were conducted with Varimax rotation to identify the motives of story updates on the three platforms, i.e., Facebook, Instagram, and WhatsApp. Items with extremely low commonalities, items that failed to load on any factors, and items that failed to fulfil the Kaiser criterion, i.e., eigenvalue >1, were removed from the analysis for all three platforms. The factor analyses explained 67.17% variance for Facebook, 66.49% for Instagram and 65.94% for WhatsApp (Table 1).

**Table 1:** Descriptive Statistics, factor and reliability analyses for study variables.

Motives	WhatsApp			Instagram			Facebook		
	Mean	S.D	Load	Mean	S.D	Load	Mean	S.D	Load
**Socially rewarding self-promotion**									
To get more likes	2.60	1.45	.830	2.56	1.46	.833	2.62	1.47	.799
To get more comments	2.51	1.42	.819	2.46	1.40	.817	2.49	1.42	.796
To become popular	2.66	1.46	.730	2.63	1.46	.753	2.65	1.47	.736
To show off	2.24	1.38	.728	2.20	1.38	.720	2.27	1.42	.728
To promote myself	2.79	1.44	.678	2.77	1.45	.706	2.75	1.46	.682
To get attention	2.93	1.38	.503	2.92	1.39	.542	2.94	1.39	.512
Eigenvalue	14.19			14.47			14.89		
% of variance explained	37.34			38.09			39.18		
Cronbach's α	.917			.914			.918		
**Social Sharing**									
To thank people	3.87	1.20	.780	3.82	1.22	.787	3.81	1.25	.802
To let people know I care about them	3.42	1.32	.747	3.46	1.34	.773	3.44	1.33	.775
To show others encouragement	4.01	1.07	.727	3.49	1.37	.753	3.45	1.37	.744
To remember special events	3.47	1.35	.726	3.96	1.12	.706	3.95	1.14	.725
To show others that Im concerned about them	3.20	1.38	.692	3.23	1.40	.699	3.20	1.39	.630
Eigenvalue	3.17			3.27			1.86		
% of variance explained	8.34			8.61			4.91		
Cronbach's α	.856			.863			.859		
**Social influence**									
Friends appreciate it	3.13	1.31	.759	3.12	1.35	.717	3.11	1.35	.770
Updating story is common now	3.68	1.33	.719	3.70	1.36	.690	3.64	1.37	.709
My friends do it	2.68	1.42	.712	2.63	1.41	.661	2.70	1.41	.682
Because everyone does it	2.58	1.36	.660	2.53	1.33	.605	2.57	1.36	.646
Friends message me	3.22	1.36	.608	3.22	1.39	.584	3.21	1.39	.600
Eigenvalue	1.95			1.70			3.23		
% of variance explained	5.14			4.48			8.51		
Cronbach's α	.834			.826			.883		
**Disclosure**									
To disclose more about my self	3.07	1.22	.810	2.98	1.26	.815	2.97	1.28	.809
To disclose more about others and me	3.04	1.25	.752	3.01	1.23	.772	2.99	1.25	.755
To share my life events with others	3.54	1.22	.674	3.49	1.26	.669	3.51	1.29	.677
To disclose happenings around me	3.53	1.20	.655	3.43	1.26	.637	3.43	1.26	.672
To provide personal information	2.54	1.25	.646	2.48	1.24	.612	2.51	1.26	.649
Eigenvalue	1.74			1.85			1.64		
% of variance explained	4.59			4.87			4.31		
Cronbach's α	.858			.856			.865		
**Escape**									
To escape from reality	2.50	1.40	.739	2.58	1.41	.775	2.53	1.41	.755
To kill time	2.87	1.47	.643	2.92	1.47	.646	2.92	1.49	.637
To forget about my problems	2.90	1.46	.621	2.89	1.46	.627	2.87	1.46	.626
To avoid loneliness	3.00	1.46	.595	2.99	1.47	.602	2.96	1.48	.589
Eigenvalue	1.63			1.60			1.55		
% of variance explained	4.28			4.22			4.08		
Cronbach's α	.787			.773			.777		
**Entertainment**									
It is fun	3.58	1.28	.715	3.59	1.29	.719	3.52	1.32	.710
Because its entertaining	3.58	1.28	.706	3.58	1.30	.691	3.55	1.31	.689
Updating story is enjoyable	3.18	1.35	.609	3.13	1.39	.546	3.13	1.37	.576
Eigenvalue	1.29			1.32			1.29		
% of variance explained	3.41			2.72			3.40		
Cronbach's α	.803			.850			.815		
**Trendy fashion**									
Updating story is now a fashion	3.03	1.47	.582	3.05	1.46	.605	3.01	1.47	.590
It is a new trend	3.19	1.46	.544	3.18	1.47	.586	3.19	1.46	.547
Updating story is cool	2.93	1.38	.506	2.92	1.39	.565	2.94	1.39	.545
Eigenvalue	1.07			1.03			1.04		.
% of variance explained	2.81			3.47			2.75		
Cronbach's α	.853			.803			.860		

Total Variance Explained = WhatsApp = 65.94 %; Instagram = 66.49%; Facebook = 67.17%.

#### 3.2.4 Intensity of story updates

To measure the intensity of the story updates on Facebook, Instagram and WhatsApp, we used four items adapted from Ellison et al. ‘s (2007) Facebook intensity scale. The items were modified according to the current study as follows: “Updating story is part of my everyday activity; “updating story has become part of my daily routine”, “I feel out of touch when I haven't updated story for a while”, “I would be sorry if story update option is unavailable”. The participants were asked to express their intensity of story updates for each platform separately. The internal reliability of the measures was good for Facebook (α = 0.83) and Instagram (α = 0.85) and excellent (α = 0.91) for WhatsApp.

#### 3.2.5 Demographics

The survey participants answered questions about their basic demographic details, such as age and gender.

### 3.3 Data analysis

The data is analysed with SPSS 23.0. Normality check (Ghasemi and Zahediasl, 2012) indicated that data is normally distributed and free from outliers and hence can be proceeded for parametric tests. We used principal component analysis with Varimax rotation to identify the different motivations behind updating Stories. To locate the cross-platform difference in updating stories and intensity of story updates among the three platforms, we have employed repeated measures of ANOVA. We also used Pearson product-moment correlation for each platform separately to examine the relationship among motivations behind updating Stories, Intensity of updating Stories, and demographic variables. Finally, we ran three separate hierarchical regression models to explore how the motivations, life position indicators, and narcissism predict the intensity of story updates on each platform.

## 4 Results

Exploratory factor analysis performed to locate the motivations behind story updates identified seven factors: socially rewarding self-promotion, social sharing, social influence, disclosure, escape, entertainment, and trendy fashion.

The first factor, *socially rewarding self-promotion*, taps into users' intention to get popularity and attention among their social circle and get social rewards in the form of likes or comments. The socially rewarding self-promotion via updating Stories on social media platforms also helps strengthen relationships with family and friends. The second gratification, *social-sharing,* reflects users’ attitude toward sharing their problems and important events in their life, i.e., birthday, anniversaries, etc., with their contacts. Updating such events as a story on social media platforms is one of the easiest ways to reach out to many people. The third motive*, social influence*, emerged from this study, indicating that social media story updates are influenced by peer pressure. The fourth motive reflects how people use the story update feature of social media platforms to disclose what is happening around them. *Escapism* has emerged as another important gratification that users seek from updating their Stories, indicating how people use the story updating feature to get a break from their everyday life or reduce stress. Updating Stories can lead to comments or likes that can further initiate a conversation and help people to divert their attention to somewhere else for a while. The sixth motivation that evolved from the current study is how people use story update features on social media platforms as entertainment. The final gratification, trendy *fashion,* reflects that people have conceived updating Stories on social media as fashionable and following that trend.

### 4.1 Cross-platform differences

The second research question inquired into the difference between WhatsApp, Instagram, and Facebook regarding motivations to update Stories and the intensity of updating Stories. To answer this research question, the intensity of updating Stories and the motivations for story updates were submitted to three platform repeated measures of ANOVA. Results are presented in Table 2.

**Table 2:** Repeated measures of ANOVA.

Variable	Whatsapp	Instagram	Facebook	ANOVA results
Intensity of update	2.96 (1.10)	2.75 (1.28)	2.68 (1.29)	F (2,674) = 5.20, p < .01, η^2^_p_ = .01
Socially rewarding self-promotion	3.23 (1.61)	3.66 (1.42)	4.11 (1.61)	F (2,674) = 4.43, p < .001, η^2^_p_ = .12
Social Sharing	3.59 (1.01)	3.12 (1.14)	2.96 (1.05)	F (2,674) = 0.06, p < .001, η^2^_p_ = .07
Social influence	3.95 (1.34)	3.54 (1.05)	3.04 (1.07)	F (2,674) = 0.02, p < .001, η^2^_p_ = .13
Disclosure	3.14 (0.97)	3.47 (0.99)	3.78 (1.02)	F (2,674) = 0.55, p < .001, η^2^_p_ = .23
Escape	2.81 (1.11)	2.84 (1.12)	2.81 (1.23)	F (2,674) = 0.06, *ns*
Entertainment	3.44 (1.10)	3.43 (1.12)	3.40 (1.14)	F (2,674) = 0.14, *ns*
Trendy fashion	3.04 (1.26)	3.57 (1.47)	3.34 (1.31)	F (2,674) = 0.01, p < .01, η^2^_p_ = .08

From the result, it was seen that the Stories update intensity is significantly different across the platforms. Participants expressed the highest intensity to update story on WhatsApp (M = 2.96, SD = 1.10), Followed by Instagram (M = 2.75, SD = 1.28) and Facebook (M = 2.68, SD = 1.29), respectively F (2,674) = 5.20, p < .01, η^2^_p_ = .01. Pairwise comparison showed that the difference between the intensity of story updates across all three platforms was significant (p < .05).

The result further showed that all the story update motivations except escape and entertainment were significantly different across the three platforms. Analysis of the mean difference (Table 2) further indicates that participants with a motive of socially rewarding self-promotion prefer to update their Stories on Facebook, followed by Instagram. In contrast, those seeking social sharing gratification prefer to update their Stories on WhatsApp, followed by Instagram and Facebook. Regarding disclosure, Facebook leads, followed by Instagram and then WhatsApp. Instagram takes the lead for trendy fashion, followed by Facebook and WhatsApp.

### 4.2 Relationships between story update motives and demographics

Three separate Pearson product-moment correlations were conducted (Refer Appendix-1, 2 &3) to identify the relationship between story update motives and age among the three platforms. The correlation between age and story update motives for WhatsApp revealed that age has a significant positive correlation with socially rewarding self-promotion (*r* = .12), social sharing (*r* = .11) and trendy fashion (*r* = .10). This finding indicates that older WhatsApp users tend to use the story update feature as a means to achieve socially rewarding self-promotion and a way for social sharing. No significant relationship between age and other WhatsApp story update gratifications was found. Similarly, in the case of Instagram, age is positively related to only two motives of story updates, i.e., socially rewarding self-promotion (*r* = .12) and social sharing (*r* = .11). The correlation results between Facebook story updates and age revealed a significant positive relationship with only social sharing motive (*r* = .01), indicating older Facebook users update their Stories to share the happenings in their lives with others.

Three separate independent sample t-tests were conducted to locate the gender difference in story updates for the three platforms. The result exhibited a significant gender difference in the socially rewarding self-promotion motivation of story updates for WhatsApp. The result suggested that females seek more socially rewarding self-promotion (t = 2.97, p < .01, M = 2.84, SD = 1.18 vs M = 2.45, SD = 1.17) gratification from story updates than males. When it comes to Instagram story updates, female users tend to seek more socially rewarding self-promotion (t = 4.31, p < .001, M = 2.97, SD = 1.19 vs M = 2.39, SD = 1.16) and trendy fashion gratifications (t = 2.09, p < .05, M = 3.25, SD = 1.19 vs M = 2.94, SD = 1.29) compared to their male counterparts. Finally, similar to WhatsApp and Instagram, there is a gender difference observed in updating Stories on Facebook. The study result revealed that female users tend to seek socially rewarding self-promotion (t = 4.04, p < .001, M = 2.95, SD = 1.19 vs M = 2.41, SD = 1.18), social influence (t = 2.16, p < .05, M = 3.20, SD = 0.98 vs M = 2.94, SD = 1.11), disclosure (t = 1.98, p < .05, M = 3.20, SD = 0.98 vs M = 2.94, SD = 1.1), and escape (t = 2.14, p < .05, M = 2.98, SD = 1.08 vs M = 2.71, SD = 1.16) gratifications by updating their Stories on Facebook.

### 4.3 Life position indicators, narcissism and motives as predictors of story update intensity

The third research question explores how the Story updates’ motivations, life position indicators, and narcissism predict the intensity of story updates on each platform. To answer this research question, we ran three separate hierarchical regression models. All the regression models included the story update intensity as a criterion variable, the demographic variables such as age and gender were entered as predictors in the first block, life position indicators in the second block, narcissism in the third block and story update motivations in the final block. The multicollinearity diagnostics found that tolerance scores and variance inflation factors (VIF) were within the acceptable limit. The summary of the regression results is presented in Table 3.

**Table 3:** Hierarchical Regression results for the relationship between seven use motivations, life position indicators, narcissism and the intensity of story updates on WhatsApp, Instagram and Facebook.

Predictors	WhatsApp	Instagram	Facebook
	β	β	β
**Block 1**			
Gender	.018	−.071	−.095
Age	.059	.013	.015
R^2^	.003	.006	.010
**Block 2**			
Interpersonal interaction	.221∗∗∗	.190∗∗	.204∗∗
Life satisfaction	−.067	−.061	−.063
Social activity	.209∗∗	.138∗	.180∗∗
ΔR^2^	.107	.065	.093
**Block 3**			
Narcissism	.277∗∗∗	.291∗∗∗	.302∗∗∗
ΔR^2^	.183	.147	.182
**Block 4**			
Socially rewarding self-promotion	.124	.133∗	.185∗∗
Social Sharing	.228∗∗∗	.126∗∗	.060
Social influence	.272∗∗∗	.308∗∗∗	.323∗∗∗
Disclosure	.202∗∗∗	.215∗∗∗	.228∗∗∗
Escape	.103	.095	.102
Entertainment	−.054	−.095	−.101
Trendy fashion	−.053	.010∗	−.039
ΔR^2^	.531	.460	.486
Total Adjusted ΔR^2^	.512	.438	.466

Note. ∗∗∗P < .001, ∗∗P < .01, ∗P < .05, ^+^P < .05.

The result revealed that demographic variables were not significant predictors of the intensity of story updates on all three platforms. When the life position indicators were added in the second block, the percentage of variance explained ranged between 6–11% for the three platforms. Interpersonal interaction and social activity positively predicted the intensity of story updates for WhatsApp, Instagram and Facebook, whereas life satisfaction negatively predicted the intensity of story updates for all the three platforms. When narcissism was added to the model, the overall percentage of the variance spiked between 28–30% for the three platforms. Narcissism has emerged as a positive predictor of the intensity of story updates for all three platforms. The final regression model with story update motivations explained the variance between 44–51% for the three platforms. Among the motives, social sharing, social influence, and disclosure significantly predicted the story update intensity for WhatsApp. In the case of Instagram, socially rewarding self-promotion, social sharing, social influence, disclosure, and trendy fashion were significant predictors of story update intensity. Finally, socially rewarding self-promotion, social influence, and disclosure gratifications were significant predictors of the intensity of story updates on Facebook.

## 5 Discussion

Although updating Stories on social media has become the order of the day, no research has been conducted yet to identify the motivations behind this activity. The present research tried to fill this void by conducting an empirical study with 338 participants to locate the various motivations behind updating Stories on three social media platforms: WhatsApp, Instagram and Facebook. Exploratory factor analyses identified seven motivations for updating Stories on the three platforms, i.e. socially rewarding self-promotion, social sharing, social influence, disclosure, escape, entertainment, and trendy fashion. These findings are in accordance with the similar motivations found for using Facebook (Quan-Haase and Young, 2010; Malik et al., 2016; Menon and Meghana, 2021) Instagram (Sheldon and Bryant, 2016; Sheldon et al., 2017; Sheldon et al., 2021a, Sheldon et al., 2021b), and WhatsApp (Karapanos et al., 2016; Bautista and Lin, 2017; Arruda Filho et al., 2021). Hence this finding supports the argument of Erz et al. (2018) that platform usage motives are antecedents of specific feature use.

Socially rewarding self-promotion has emerged as a major motive behind updating Stories on all three platforms. This finding indicates that users update Stories with an intention to promote themselves and get rewards in the form of likes and comments. The Stories updates feature in Facebook allows users to directly like and comment on others' Stories by clicking on the specific ‘like’ or ‘comment’ options. On the other hand, Instagram and WhatsApp allow their users to like or react to others' updates via emojis or direct comments. In the age of social media networking, social rewards and self-promotion help people increase and widen social capital (Ellison et al., 2007; Urista et al., 2009). For instance, getting appreciation has been identified as one of the important motivations behind sharing photos on Flickr (Malinen, 2011) and Facebook (Malik et al., 2016). Our result corroborates the findings of Sheldon and Bryant's (2016) study, in which they found self-promotion is one of the important gratification sought from Instagram. In the latest study, Scherr and Wang (2021) identified socially rewarding self-presentation as one of the main motivations behind TikTok usage.

Social sharing was another motivation for updating Stories. People often use Stories as a tool to communicate with others and share their happiness and sorrows with their social circle. The story feature allows users to effectively mirror thoughts in their minds through pictures, videos, or both. For instance, users can share their birthday celebration in the form of a Story or an achievement in their life with others in their friends’ circle to get their attention. Similarly, users can express their gratitude to their beloved ones by updating a Story to seek affection or thank them. Many studies (Malik et al., 2016; Oeldorf-Hirsch and Sundar, 2016; Alhabash and Ma, 2017) have identified social sharing as one of the important motivations behind social media platform usage.

Past studies have revealed that social influence motivates people to adopt certain new practices and behaviours on social media platforms. For instance, Dhir and Torsheim (2016) & Dhir et al. (2017) identified that social influence motivates people to tag photos online on Facebook. Our study indicates social influences such as peer pressure or following a particular trend motivate people to update Stories on social media platforms. This result upheld the argument of Workman (2014) that social influence is an important determinant behind the adoption of new media technologies and their features.

Most social media platforms are designed and developed in such a way to motivate their users to disclose information about themselves and other people around them. Prior literature has recognised disclosure as one of the prominent motives behind using various social media (Papacharissi and Mendelson, 2010; Hollenbaugh and Ferris, 2014; Lin and Chu, 2021). Despite increasing concern over disclosing information on social media platforms (Fogel and Nehmad, 2009; Orito et al., 2014; Malik et al., 2016), our study result interestingly found that disclosure is one of the important motivations for updating Stories on social media platforms. This finding is in line with the prior literature that suggests users share private and sensitive information on social media platforms (Hargittai, 2007; Malik et al., 2016) and is explained with the aid of the ‘privacy paradox’ (Barnes, 2006; Kokolakis, 2017). Hence from the finding, it could be inferred that since ‘Story’ is a new feature and the easiest way of sharing and communicating one's emotions, users prefer this feature to disclose personal and sensitive information in a series of photos or videos. Hence the Story feature is one of the easiest and thrifty ways to express oneself, family, peers and activities to one's social circle as well as the general public in the form of a mere 24 h duration, short-living collage of images and videos.

Sundar and Limperos (2013) argues that even though medium-specific gratifications are emerging from new media studies, certain gratifications of traditional media are still prevalent in new media research. The current study underscores this argument by identifying two traditional gratification motives, i.e. escape and entertainment for updating Stories on social media platforms. The recent studies support this finding where Menon and Meghana (2021) identified escape as one motivation behind using Facebook. Sheldon and Newman (2019) and Sheldon et al., 2021a, Sheldon et al., 2021b located escape as one of the motives for Instagram usage. Similarly, recent studies (Alhabash and Ma, 2017; Meng and Leung, 2021) also identified entertainment as one of the principal motivations for social media platform use. From this finding, we can infer that people update Stories on social media to escape from reality or for some entertainment. For example, updating Stories will get comments from other users, leading to interpersonal communication via chats. These chats may entertain the users or temporarily divert their attention from their present state of mind.

Finally, trendy fashion was identified as another motivation for updating Stories on social media platforms. As per Sundar and Limperos (2013), technological affordances lead to certain medium-specific or content specific gratifications. The sense of experiencing novelty and following particular trends has always driven social media usage ever since its existence (Papacharissi and Mendelson, 2010; Scherr and Wang, 2021). Therefore it is not surprising that trendy fashion was one of the motivations behind updating Stories on social media platforms. Further, the social influence and trendy fashion exhibited high positive correlations among all the three platforms indicating factors such as peer pressure influence people to follow the trend of updating Stories. Also, prior studies have identified coolness (Sundar et al., 2014; Sheldon and Bryant, 2016) and trendiness (Scherr and Wang, 2021) as motivations behind using social media platforms.

The cross platforms analysis of the three platforms indicated a significant difference in their intensity of usage. The result showed that WhatsApp has the highest intensity of story updates, followed by Instagram and Facebook. This finding is in tune with the recent reports on social media usage in India that WhatsApp has higher usage in India, followed by Instagram and Facebook (Chakravarti, 2021; Keelery, 2021a).

The cross-platform analysis (see Table 2) further showed a significant difference in all the motivations to update Stories on the three platforms under study. The result revealed that the motivations to update Stories on WhatsApp are social influence, social sharing, entertainment, socially rewarding self-promotion, trendy fashion, and escape, respectively. On the other hand, the most prominent motivation to update Stories on Instagram is socially rewarding self-promotion, followed by trendy fashion, social influence and disclosure. As for Facebook, we see that after socially rewarding self-promotion and disclosure, entertainment, trendy fashion, social influence, social sharing, and escape followed, respectively (See Table 4). This finding is in line with Alhabash and Ma's (2017) cross-platform analysis of the motivations to use four platforms, i.e. Facebook, Instagram, Twitter and Snapchat.

**Table 4:** Ranking of motivations to update Stories across WhatsApp, Instagram and Facebook.

	Whatsapp	Instagram	Facebook
1	Social influence	Socially rewarding self-promotion	Socially rewarding self-promotion
2	Social Sharing	Trendy fashion	Disclosure
3	Entertainment	Social influence	Entertainment
4	Socially rewarding self-promotion	Disclosure	Trendy fashion
5	Disclosure	Entertainment	Social influence
6	Trendy fashion	Social Sharing	Social Sharing
7	Escape	Escape	Escape

The study results also revealed some interesting relationships between age, gender and Story updating motivations. The study result suggests that the level of socially rewarding self-promotion, social sharing and trendy fashion increases with age for WhatsApp story updates. Similarly, socially rewarding self-promotion and social sharing gratifications increase with age for Instagram users. In the case of Facebook, as compared to younger users, older users seek higher longing for social sharing gratification from updating Stories. Our finding aligns with the past studies on social media (Malik et al., 2016; Scherr and Wang, 2021), which located that gratifications sought from social media platforms depend on age. Malik et al. (2016) suggest that the possible reason behind this age dependence on gratifications could be the difference in the level of awareness and degree of influence of technology between the two user groups.

The result also highlights gender differences in the motivations for updating Stories on all three platforms. In the case of WhatsApp, female users tend to seek more socially rewarding self-promotion than males by updating Stories. Again, when it comes to Instagram story updates, females exhibited an inclination towards socially rewarding self-promotion and trendy fashion gratifications. The study result also revealed that female users seek socially rewarding self-promotion, social influence, disclosure, and escape gratifications by updating their Stories on Facebook. This finding is in line with previous studies conducted on social media uses and gratifications (Smock et al., 2011; Krause et al., 2014; Malik et al., 2016; Scherr and Wang, 2021; Menon and Meghana, 2021) that showed significant gender differences existed in the gratification motives for social media platform usage.

The final research question of our study was intended to identify the predictors of the intensity of Stories updates for WhatsApp, Instagram and Facebook. Consistent with the findings of previous scholarships on social media platforms (Carpenter, 2012; Skues et al., 2012; Davenport et al., 2014; Sheldon and Bryant, 2016; Sheldon et al., 2021a, Sheldon et al., 2021b), we included contextual age and narcissism as predictors of the intensity of updating Stories. The result revealed that interpersonal interaction and social activity positively predicted the intensity of updating Stories on all three platforms. That means socially active people with a higher sense of interpersonal interaction are more prone to update Stories on social media platforms. On similar lines, our result indicated that narcissists were keen on updating Stories on WhatsApp, Instagram and Facebook. These findings corroborate the results of Sheldon and Bryant (2016), which identified that narcissists spent more time editing photos on Instagram. Kristinsdottir et al. (2021), in their recent study, found that narcissists exhibit a higher intensity of social media usage. The probable reason for the social media behaviours and narcissism could be the usage of ‘selfies’ (Weiser, 2015). Even selfies dominate the social media Stories uploaded by people. Hence it is plausible to assume that narcissists exhibit higher intensity in updating their social media Stories.

The current study revealed that among the motives of social media story updates, social influence and disclosure positively predicted the intensity of updating Stories on all three platforms. This finding is supported by prior studies on social media platforms that recognise disclosure (Hollenbaugh and Ferris, 2014; Malik et al., 2016; Al-Kandari et al., 2016) and social influence (Malik et al., 2016; Sherman et al., 2018) as important gratifications. During our focus group interview, most people revealed that they started updating Stories on social media platforms after seeing their friends doing so. Also, participants reported that updating Stories is one of the easiest ways to disclose important events in their lives. Posting photos and videos of time-bound events such as celebrations and anniversaries as Stories will communicate the messages to their contacts and followers. Since these Stories have a lifetime of 24 h, the message will be automatically deleted.

Socially rewarding self-promotion emerged as a positive predictor of Stories updates on Instagram and Facebook. That means users update their Stories on Instagram and Facebook to get more likes and comments and thus become popular among their friends' circle and peer groups (Malik et al., 2016; Sheldon and Bryant, 2016). Many social media influencers and celebrities nowadays repost their shared video and photographs as Stories. This will help them reach out to their followers easily and make them watch the main content. For example, people often post Instagram reels as their story update, which will notify their followers that a new reel has been posted. It is important to note that socially rewarding self-promotion did not predict the intensity of updating Stories on WhatsApp. This is probably because what app is more of a personal messaging platform than a social networking application. That means social media influencers’ WhatsApp story updates can only be seen by their close contacts or saved numbers on their phone, whereas story updates on Facebook and Instagram can be seen by millions of followers.

Social sharing predicted the intensity of updating Stories on WhatsApp and Instagram, indicating the intimacy of users with WhatsApp and Instagram. These two platforms are largely used for personal communication. This finding supports the argument of Quan-Haase and Young (2010) that people uses instant messaging applications for more intimate conversations, share their problems, and receive social and emotional support from their peer groups. Hence it is plausible that people prefer to use the Stories feature as a tool for effectively expressing their emotions and empathy towards others using WhatsApp and Instagram. Consistent with prior literature (Sundar et al., 2014), trendy fashion became a positive predictor of Instagram story updates. Sundar et al. (2014) suggested that platform-centred affordances such as ‘coolness’ persuade users to experiment with new things and adopt new features and behaviours. Further, recent studies on Instagram (Sheldon and Bryant, 2016)and social media platforms (Scherr and Wang, 2021) located coolness and trendy fashion as key predictors of user behaviours.

### 5.1 Theoretical and practical contributions

First, this study extended the application of the Uses and gratification theory beyond traditional media to explicate the phenomenon of updating Stories on social media platforms. Uses and gratification is a popular theoretical lens widely used by researchers to locate the motivations behind the usage of various mass media. But the current study introduced a new dimension to this theory by applying it to identify the motivations behind using the particular feature, i.e. ‘Story’, on social media platforms. With the uses and gratifications theory, the current study identified seven motivations behind updating Stories on social media. Five of the seven motives, i.e. entertainment, escape, social influence, social sharing and disclosure, were the same as prior uses and gratifications of social media. Two new motives: socially rewarding self-promotion and trendy fashion, emerged from the current research, probably unique to Story updates. Thus current research confirmed Sundar and Limperos' (2015) suggestion that new media exhibit certain medium-specific gratifications along with traditional gratifications.

Second, this study contributes to the existing literature by identifying the importance of social-psychological predictors in uses and gratifications. The current research endorses the prior scholarship (Papacharissi and Mendelson, 2010; Sheldon and Bryant, 2016) by understanding which contextual age indicators best predict the intensity of updating Stories on three platforms, i.e. WhatsApp, Instagram and Facebook. Consistent with the prior literature (Sheldon and Bryant, 2016; Andreassen et al., 2017; Barry and McDougall, 2018; Meng and Leung, 2021), this study also found psychological predictors such as narcissism play an important role in social media behaviours.

Finally, by identifying two modalities-based gratifications (Sundar et al., 2014; Sundar and Limperos, 2013), i.e., trendy fashion and socially rewarding self-promotion, we empirically confirmed that platform centred affordances could influence media behaviours (Rathnayake and Winter, 2018). Hence, we state that if the researchers can situate a ‘modernised’ approach in line with the booming new media technologies, Uses and gratification is still a relevant and axiomatic theory (Lin, 2006) that can predict user behaviours.

Besides its theoretical implications, the study has certain implications for the practice. Nowadays, social media platforms play an important role in organisations and businesses, and social media marketing is at its pinnacle (Sheldon et al., 2017; Chen, 2018; Evans et al., 2021). Business firms, multinational companies, celebrities and social media influencers heavily depend on social media platforms for publicity and promotion. By locating user motivations from a theoretical perspective, the current study provides guiding insights to media influencers in designing proper PR strategies to communicate effectively with their targeted audiences. For example, companies can use poll stickers or question stickers as Stories to get customer feedback. Recent media reports (Rayes, 2019) say that OTT platforms like Netflix are developing Story-like features for their marketing purpose. Among the three platforms, Instagram and Facebook Stories features were largely used for marketing, whereas small business firms and direct marketers use WhatsApp Story features. WhatsApp status Stories are the easiest way for business promotion without clogging the user with messages.

### 5.2 Limitations and future research

Like any other research, this study is not free from limitations, and those limitations set grounds for further investigation. Firstly the current study utilised samples from a single country. Hence caution must be taken while generalising the results to other cultural settings. We request future researchers to conduct a similar study with larger samples in another country. Additionally, the current research relied heavily on self-reported data, subject to desirability bias and limiting external validity. Secondly, we have used only three components of contextual age to predict the intensity of Stories updates. We recommend that future researchers consider economic security, physical health, and mobility while measuring the life position indicators. Lastly, we have used only one psychological predictor, i.e. narcissism, to predict the Story updates. Recent studies (Sheldon et al., 2021a, Sheldon et al., 2021b; Meng and Leung, 2021) showed that psychological factors such as FOMO and the big five personality traits influence social media engagement behaviours. Hence we suggest future researchers include these factors in their study.

## Declarations

### Author contribution statement

Devadas Menon: Conceived and designed the experiments; Performed the experiments; Analyzed and interpreted the data; Contributed reagents, materials, analysis tools or data; Wrote the paper.

### Funding statement

This research did not receive any specific grant from funding agencies in the public, commercial, or not-for-profit sectors.

### Data availability statement

Data will be made available on request.

### Declaration of interests statement

The authors declare no conflict of interest.

### Additional information

No additional information is available for this paper.

## Figures and Tables

**Appendix – 1:** Pearson correlation between the variables of Whatsapp status updates

	1	2	3	4	5	6	7	8	9	10	11	12	13	14
1. Socially rewarding self-promotion	-													
2. Social Sharing	.367∗∗	-												
3. Social influence	.658∗∗	.428∗∗	-											
4. Disclosure	.518∗∗	.461∗∗	.513∗∗	-										
5. Escape	.574∗∗	.337∗∗	.568∗∗	.364∗∗	-									
6. Entertainment	.529∗∗	.437∗∗	.585∗∗	.503∗∗	.449∗∗	-								
7. Trendy fashion	.735∗∗	.341∗∗	.761∗∗	.487∗∗	.558∗∗	.613∗∗	-							
8. Interpersonal interaction	.147∗∗	.219∗∗	.090	.272∗∗	.057	.175∗∗	.143∗∗	-						
9. Life satisfaction	.077	.230∗∗	.080	.219∗∗	−.076	.098	.097	.445∗∗	-					
10. Social activity	.181∗∗	.248∗∗	.125∗	.284∗∗	.041	.153∗∗	.122∗	.415∗∗	.504∗∗	-				
11. Narcissism	.378∗∗	.052	.249∗∗	.193∗∗	.368∗∗	.167∗∗	.300∗∗	.027	.001	.067	-			
12. Intensity of status updates	.520∗∗	.519∗∗	.457∗∗	.556∗∗	.451∗∗	.273∗	.360∗	.279∗∗	.140∗	.262∗∗	.290∗∗	-		
13. Age	.120∗	.119∗	.060	.058	−.067	−.021	.109∗	.182∗	.107∗	.143∗∗	−.037	.056	-	
14. Gender	−.160∗∗	.104	−.069	−.086	−.064	.087	−.054	.011	.083	−.133∗∗	−.088	.007	−.183∗∗	-

**Appendix – 2:** Pearson correlation between the variables of Instagram status updates

	1	2	3	4	5	6	7	8	9	10	11	12	13	14
1. Socially rewarding self-promotion	-													
2. Social Sharing	.366∗∗	-												
3. Social influence	.676∗∗	.451∗∗	-											
4. Disclosure	.514∗∗	.496∗∗	.533∗∗	-										
5. Escape	.555∗∗	.321∗∗	.548∗∗	.359∗∗	-									
6. Entertainment	.542∗∗	.464∗∗	.612∗∗	.513∗∗	.455∗∗	-								
7. Trendy fashion	.750∗∗	.340∗∗	.759∗∗	.492∗∗	.539∗∗	.617∗∗	-							
8. Interpersonal interaction	.219∗∗	.228∗∗	.164∗∗	.317∗∗	.045	.219∗∗	.211∗∗	-						
9. Life satisfaction	.173∗∗	.253∗∗	.130∗	.252∗∗	−.069	.148∗∗	.160∗∗	.488∗∗	-					
10. Social activity	.235∗∗	.253∗∗	.203∗∗	.328∗∗	.045	.176∗∗	.172∗∗	.454∗∗	.542∗∗	-				
11. Narcissism	.359∗∗	.088	.284∗∗	.193∗∗	.378∗∗	.161∗∗	.296∗∗	.065	.004	.067	-			
12. Intensity of status updates	.507∗∗	.420∗∗	.456∗∗	.524∗∗	.442∗∗	.160∗	.370∗∗	.216∗∗	.099	.194∗∗	.312∗∗	-		
13. Age	.120∗	.113∗	.048	.078	−.091	−.023	.104	.194∗∗	.116∗	.169∗∗	−.045	.034	-	
14. Gender	−.229∗∗	.085	−.094	−.082	−.105	.073	−.114∗	−.009	.051	−.167∗∗	−.115∗	−.075	−.296∗∗	-

**Appendix – 3:** Pearson correlation between the variables of Facebook status updates

	1	2	3	4	5	6	7	8	9	10	11	12	13	14
1. Socially rewarding self-promotion	-													
2. Social Sharing	.390∗∗	-												
3. Social influence	.694∗∗	.414∗∗	-											
4. Disclosure	.525∗∗	.499∗∗	.524∗∗	-										
5. Escape	.592∗∗	.340∗∗	.598∗∗	.392∗∗	-									
6. Entertainment	.571∗∗	.451∗∗	.624∗∗	.519∗∗	.490∗∗	-								
7. Trendy fashion	.745∗∗	.361∗∗	.760∗∗	.492∗∗	.567∗∗	.617∗∗	-							
8. Interpersonal interaction	.170∗∗	.241∗∗	.116∗∗	.320∗∗	.060	.207∗∗	.158∗∗	-						
9. Life satisfaction	.072	.257∗∗	.034	.234∗∗	−.082	.070	.067	.464∗∗	-					
10. Social activity	. 196∗∗	.269∗∗	.166∗∗	.325∗∗	.068	.184∗∗	.164∗∗	.436∗∗	.542∗∗	-				
11. Narcissism	.374∗∗	.083	.309∗∗	.214∗∗	.387∗∗	.209∗∗	.316∗∗	.026	−.048	.038	-			
12. Intensity of status updates	.548∗∗	.402∗∗	.462∗∗	.551∗∗	.455∗∗	.178∗	.132∗	.249∗∗	.123∗	.242∗∗	.319∗∗	-		
13. Age	.089	.018∗	.028	.034	−.100	−.043	.047	.169∗∗	.106	.138∗	−.044	.040	-	
14. Gender	−.216∗∗	.068	−.117∗	−.108	−.166∗	.060	−.094	−.016	.043	−.160∗∗	−.087	−.099	−.256∗∗	-

## Appendix ASupplementary data

Questionnaire.docx Questionnaire.docx
