# Supplementary material for: Updating ‘Stories’ on social media and its relationships to contextual age and narcissism: A tale of three platforms – WhatsApp, Instagram and Facebook
Source: Heliyon. 2022 May 14;8(5):e09412. doi: 10.1016/j.heliyon.2022.e09412 (PMC9119837; doi:10.1016/j.heliyon.2022.e09412)
Supplement: Questionnaire.docx [file mmc1.docx]

**Questionnaire**

**Updating ‘Stories’ on Social Media and its relationship with life position indicators and personality traits: A study on WhatsApp, Instagram and Facebook**

Dear respondent,

We are conducting a study on updating ‘Stories’ (status) on social media platforms and its relationship with life position indicators and personality traits. This research aims to identify the motivations behind updating ‘stories’ on the three social media platforms, i. e. Facebook, Instagram and WhatsApp. Besides collecting information about your demographic details and

Motives behind updating stories we also collect a few insights about your personality, life position indicators and intensity of story updates in each of the three platforms. It will take about 30 Mins to complete this survey. If you feel any discomfort in filling out the questionnaire, you are free to leave out any question(s), or you are free to quit this survey any time. We confirm that we are not collecting any personally identifiable details about you and request you not provide any personally identifying information in the questionnaire. We assure you that the responses provided will be kept confidential and not be shared with any third party. We also confirm that your data will be used only for the research purpose mentioned above and will not be used for any other purposes.

If you agree to participate in the survey, kindly click ‘**YES’** to proceed with the survey. If you do not agree, please click ‘**NO’**.

Thank you

(Researcher)

**Part I**

I have read and understood the above study’s objectives and information asked from me and have had the opportunity to ask questions. I understand that my participation is voluntary and that I am free to withdraw at any time, without giving a reason and without cost. I understand that I will be given a copy of this consent form. I voluntarily agree to take part in this study.

YES

NO

**Part II**

1. Do you update Stories on WhatsApp, Instagram and Facebook?”

YES

NO

1. “Have you updated Stories on WhatsApp, Instagram and Facebook in the last 30 days?”:

YES

NO

**Part III**

1. Your gender Male Female
2. What is your age ---------------

**Part IV**

1. **Please give your responses to the following statements on 5 point scale ranging from 1 (strongly agree ) to 5 ( Strongly disagree).**

- I get to see my friends as often as I would like
- I spend enough time in communicating with family or friends by phone or email
- I have ample opportunity to communicate with other people
- I find a great deal of happiness in my life
- I have been very successful in achieving my aims or goals in life
- I’m very content and satisfied with my life
- I often travel, take trips or vacation with others
- I often visit my friends, relatives and neighbours at their place
- I often participate in games, sports or activities with others.

**Part V**

1. **The following questions in the section deal with some insights into your personality. Please give your responses to the following statements on 5 point scale ranging from 1 (strongly agree ) to 5 ( Strongly disagree).**

- I can become entirely absorbed in thinking about my personal affairs, my health, my cares or my relations to others.
- My feelings are easily hurt by ridicule or the slighting remarks of others.
- When I enter a room, I often become self-conscious and feel that the eyes of others are upon me.
- I dislike sharing the credit of an achievement with others.
- I feel that I have enough on my hands without worrying about other people’s troubles.
- I feel that I am temperamentally different from most people.
- I often interpret the remarks of others in a personal way.
- I easily become wrapped up in my own interests and forget the existence of others.
- I dislike being with a group unless I know that I am appreciated by at least one of those present.
- I am secretly “put out” or annoyed when other people come to me with their troubles, asking me for my time and sympathy.

**Part VI**

**The following sections deal with the motives/ reasons for updating stories on Facebook, Instagram and WhatsApp. Pleased choose the platform first from the three options and then kindly provide your reason for updating stories on that platform. Please repeat the procedure for all the three platforms**

Please select the platform: Facebook Instagram WhatsApp

**Motives for updating Story**

- To disclose more about myself
- To disclose more about others and me
- To share my life events with others
- To disclose happenings around me
- To provide personal information
- To disclose my photography skills
- Because everyone does it
- My friends do it
- Friends appreciate it
- Friends message me
- Updating story is now a fashion
- It is a new trend
- It is fun
- Updating story is cool
- Updating story is very common now
- To become popular
- To get more likes
- To get more comments
- To show off
- To promote myself
- To get attention
- To avoid loneliness
- To escape from reality
- To relax
- To kill time
- Because it’s entertaining
- Updating story is enjoyable
- Updating story is part of my online activities
- Updating story is one of my habits
- To stay connected with others
- To remember special events
- To thank people
- To let people know I care about them
- To show others encouragement
- To show others that I’m concerned about them
- To forget about my problems

**Part VII**

**The following section deal with your intensity of updating stories on Facebook, Instagram and WhatsApp. Pleased choose the platform first from the three options and then kindly provide your reason for updating stories on that platform. Please repeat the procedure for all the three platforms**

- Updating story is part of my everyday activity
- updating stories has become part of my daily routine
- I feel out of touch when I haven’t updated Story for a while
- I would be sorry if the story update option is unavailable

**Thank you for your responses!**

********************
